# Phenotypic Heterogeneity of Monogenic Frontotemporal Dementia

**DOI:** 10.3389/fnagi.2015.00171

**Published:** 2015-09-01

**Authors:** Alberto Benussi, Alessandro Padovani, Barbara Borroni

**Affiliations:** ^1^Centre for Ageing Brain and Neurodegenerative Disorders, Neurology Unit, Department of Clinical and Experimental Sciences, University of Brescia, Brescia, Italy

**Keywords:** frontotemporal dementia, frontotemporal lobar degeneration, mutation, genetics, neuroimaging

## Abstract

Frontotemporal dementia (FTD) is a genetically and pathologically heterogeneous disorder characterized by personality changes, language impairment, and deficits of executive functions associated with frontal and temporal lobe degeneration. Different phenotypes have been defined on the basis of presenting clinical symptoms, i.e., the behavioral variant of FTD, the agrammatic variant of primary progressive aphasia, and the semantic variant of PPA. Some patients have an associated movement disorder, either parkinsonism, as in progressive supranuclear palsy and corticobasal syndrome, or motor neuron disease (FTD–MND). A family history of dementia is found in 40% of cases of FTD and about 10% have a clear autosomal-dominant inheritance. Genetic studies have identified several genes associated with monogenic FTD: microtubule-associated protein tau, progranulin, TAR DNA-binding protein 43, valosin-containing protein, charged multivesicular body protein 2B, fused in sarcoma, and the hexanucleotide repeat expansion in intron 1 of the chromosome 9 open reading frame 72. Patients often present with an extensive phenotypic variability, even among different members of the same kindred carrying an identical disease mutation. The objective of the present work is to review and evaluate available literature data in order to highlight recent advances in clinical, biological, and neuroimaging features of monogenic frontotemporal lobar degeneration and try to identify different mechanisms underlying the extreme phenotypic heterogeneity that characterizes this disease.

## Introduction

Frontotemporal dementia (FTD) is the second most common cause of dementia in the presenile age group (<65 years of age), and accounts for 5–15% of all cases of dementia, with a prevalence of 10–15 per 100,000 subjects in the age group of 45–65 years (Bird et al., 2003; Feldman et al., 2003; Borroni et al., 2015b).

Frontotemporal dementia is a genetically and pathologically heterogeneous disorder characterized by personality changes, language impairment, and deficits of executive functions, associated with frontal and temporal lobe degeneration (The Lund and Manchester Groups, 1994; Neary et al., 1998; McKhann et al., 2001).

Current redefined clinical criteria recognize different phenotypes on the basis of presenting clinical symptoms, namely the behavioral variant of FTD (bvFTD), the agrammatic variant of primary progressive aphasia (avPPA), and the semantic variant of PPA (svPPA) (Mesulam, 1982; Gorno-Tempini et al., 2011; Rascovsky et al., 2011). Moreover, some patients have an associated parkinsonism, as in progressive supranuclear palsy (PSP) and corticobasal syndrome (CBS), or motor neuron disease (FTD–MND) (Lomen-Hoerth et al., 2002; Burrell et al., 2011; Armstrong et al., 2013).

A family history of dementia is found in 25–50% of cases of FTD and about 10% have a clear autosomal-dominant inheritance (Seelaar et al., 2008; Rohrer et al., 2009). Genetic studies have identified several genes associated with monogenic FTD: in 1998, mutations in the microtubule-associated protein tau (*MAPT*) gene on chromosome 17 were identified in families with FTD and parkinsonism (Hutton et al., 1998; Poorkaj et al., 1998; Spillantini et al., 1998). In spite of this finding, there have been numerous reports of families with linkage to chromosome 17q21 but without evidence of *MAPT* pathogenic mutations (Froelich et al., 1997; Kertesz et al., 2000; Rosso et al., 2001; Rademakers et al., 2002).

In 2006, the second FTD-related gene on chromosome 17 was discovered in the progranulin (*GRN*) gene (Baker et al., 2006; Cruts et al., 2006), accounting for even more cases than mutations in *MAPT* explaining all the 17q21-linked autosomal-dominant FTD cases not accounted for by *MAPT* mutations (www.molgen.ua.ac.be/ADMutations/).

Eventually, a genetic linkage to a locus on chromosome 9p was found in many families with FTD and motor neuron disease (Morita et al., 2006; Vance et al., 2006; Luty et al., 2008; Le Ber et al., 2009; Boxer et al., 2011); only recently in 2011, two separate groups identified the defect as being an expanded hexanucleotide repeat in a non-coding region of the chromosome 9 open reading frame 72 (*C9orf72*) gene (DeJesus-Hernandez et al., 2011; Renton et al., 2011).

Other uncommon mutations have been identified in the valosin-containing protein (*VCP*) gene, which cause inclusion body myopathy with Paget disease of bone and FTD (Watts et al., 2004), and in the charged multivesicular body protein 2B (*CHMP2B*) gene, involved in the endosomal–lysosomal pathway (Skibinski et al., 2005). Few reports have also described FTD associated with mutations in the fused in sarcoma (*FUS*) (Broustal et al., 2010; Van Langenhove et al., 2010), dynactin (*DCTN1*) (Münch et al., 2005), sequestosome 1 (*SQSTM1*) (Fecto et al., 2011; Rubino et al., 2012; van der Zee et al., 2014), colony-stimulating factor 1 receptor (*CFS1R*) (Münch et al., 2005; Guerreiro et al., 2013b), triggering receptor expressed on myeloid cells (*TREM2*) (Borroni et al., 2013; Guerreiro, 2013), ubiquilin-2 (*UBQLN2*) (Gellera et al., 2013), and heterogeneous nuclear ribonucleoprotein A2B1 (*hnRNPA2B1*) genes (Kim et al., 2013).

Clinical FTD is associated with different types of underlying neuropathology, and the term frontotemporal lobar degeneration (FTLD), characterized by the relative selective degeneration of the frontal and temporal lobes, is used to describe the pathological hallmarks of the disease. Abnormal intracellular accumulation of insoluble proteins have been identified with immunohistochemical techniques and, according to the predominant neuronal inclusions, FTLD cases have been categorized into several types (Mackenzie et al., 2008, 2010). At first, neuronal and glial inclusions of hyperphosphorylated tau protein were identified in FTLD-tau, which represent nearly 30–40% of FTLD cases, and are found in patients carrying *MAPT* gene mutations, as well as in Pick’s disease, PSP, and corticobasal degeneration (CBD) (Josephs et al., 2006).

Only briefly after the discovery of the *GRN* gene, TAR DNA-binding protein 43 (TDP-43) inclusions were identified as the predominant hallmark of the originally identified ubiquitin positive inclusions, which were termed FTLD-U (Mackenzie et al., 2006), and subsequently renamed FTLD-TDP (Arai et al., 2006; Neumann et al., 2006). TDP-43 inclusions have also been identified in the majority of amyotrophic lateral sclerosis (ALS) and FTD-MND cases, in particular in patients with mutations of the *C9orf72* gene. Four FTLD-TDP subtypes (A–D) have been proposed based on the location of these inclusions (Mackenzie et al., 2011).

Finally in 2009, the ~10% of FTLD cases negative for tau or TDP-43 pathology were characterized by inclusions immunoreactive for FUS, which were then labeled as FTLD-FUS (Munoz et al., 2009; Neumann et al., 2009b; Rademakers et al., 2009). Furthermore, the inclusions positive for FUS were found to also stain for other members of the FET family of DNA/RNA-binding proteins, comprising the TATA-binding protein-associated factor 15 (TAF-15) and Ewing sarcoma protein (EWS), distinguishing FTLD with FUS pathology from ALS with *FUS* mutations (Neumann et al., 2011).

Patients with monogenic FTD often present with an extensive phenotypic variability, even among different members of the same kindred carrying an identical disease mutation.

The objective of the present work is to review and evaluate available literature data in order to highlight recent advances in clinical, biological, and neuroimaging features of monogenic FTLD and try to identify the hallmarks of different monogenic FTDs.

## *MAPT* Mutations

The *MAPT* gene on chromosome 17, encoding the tau protein, was the first gene identified in families with FTD and parkinsonism (Hutton et al., 1998; Poorkaj et al., 1998; Spillantini et al., 1998). Tau is a natively unfolded protein that is abundantly expressed in the central nervous system where it regulates microtubule assembly and stabilization, being involved in signal transduction and axonal transport (Goedert et al., 1989b). Forty-four pathogenic mutations in the *MAPT* gene have been identified so far (Cruts et al., 2012), causing the accumulation of hyperphosphorylated tau protein in neurons and glial cells (Hutton et al., 1998).

Alternative mRNA splicing of exons 2, 3, and 10 of the *MAPT* gene leads to the assembly of six tau isoforms that are expressed in the adult human brain (Neve et al., 1986; Goedert et al., 1989a; Andreadis et al., 1992). The tau isoforms differ from each other by the presence or absence of a 29-aminoacid or 58-aminoacid insert in the amino-terminal half and by the inclusion or not of a 31-aminoacid repeat encoded by exon 10 of *MAPT* in the carboxy-terminal half of the protein. The inclusion or exclusion of exon 10 results in the production of three isoforms with four repeats each (4R) or three isoforms with three repeats each (3R), respectively (Spillantini and Goedert, 2013). These repeats comprise the microtubule-binding domains of tau, with 4R tau having greater predisposition for microtubule assembly than 3R tau (Goedert and Jakes, 1990).

Mutations can be differentiated into missense mutations in exons 9–13, influencing the normal function of the tau protein in microtubule stabilization, and intronic and some coding mutations modifying the splicing of exon 10 at the mRNA level, resulting in a disproportionate ratio of 3R/4R tau isoforms (Goedert et al., 1989a).

The frequency of *MAPT* gene mutations is highly variable, ranging from 0–3% in sporadic FTD to 5–20% in familial cases (Houlden et al., 1999; Rizzu et al., 1999; Poorkaj et al., 2001; Binetti et al., 2003; Rademakers et al., 2004; Kaivorinne et al., 2008; Pickering-Brown et al., 2008).

Heterogeneous clinical phenotypes with high penetrance (van Herpen et al., 2003) have been observed in patients with mutations in the *MAPT* gene, even in families bearing the same pathogenic mutation (Van Swieten et al., 1999; Janssen et al., 2002; Boeve et al., 2005).

Disease onset is also highly variable, ranging between 45 and 65 years, with a mean age of onset of 55 years, nearly 10 years earlier than in patients with *GRN* mutations (Van Swieten and Spillantini, 2007). The disease may however occasionally present between 20 and 30 years (Van Swieten and Spillantini, 2007; Momeni et al., 2010) or after 70 years in a few mutations (Hayashi et al., 2002). The mean disease duration is 9 years, ranging from 5 to 20 years (Seelaar et al., 2011).

The clinical phenotype of patients with *MAPT* mutations can be divided into a dementia-dominant type and a parkinsonism-dominant type. The most common presentation, as with *GRN* mutations, is bvFTD, characterized by behavioral inhibition, impaired social behavior, and obsessive–compulsive disorder (Van Swieten and Spillantini, 2007). As with *GRN* mutations, patients may subsequently present parkinsonism, which rarely is the exclusive clinical presentation, and more often develops in association with bvFTD (Van Swieten and Spillantini, 2007; Rohrer et al., 2011b). However, some patients have a primary parkinsonian syndrome as in CBS or, more rarely, as in PSP (Dickson et al., 2007; Van Swieten and Spillantini, 2007). Parkinsonian features consist of symmetrical bradykinesia, postural instability and rigidity affecting axial and appendicular musculature, absence of resting tremor, and poor or no responsiveness to levodopa (Ghetti et al., 2015). In patients carrying the N279K mutation, parkinsonism is an early feature and asymmetric resting tremor and levodopa responsiveness have been observed (Tsuboi et al., 2002). Numerous other motor signs and symptoms have been observed, comprising supranuclear gaze palsy, upper and lower motor neuron dysfunction, dystonia, myoclonus, postural and action tremor, apraxia of eyelid opening and closing, dysphagia, and dysarthria (Ghetti et al., 2015).

Patients may also develop language impairment, which usually is not a presenting feature (Seelaar et al., 2008; Rohrer et al., 2010b); indeed the P301L mutation was recently observed in a family with impaired single word comprehension suggestive of verbal semantic impairment (Ishizuka et al., 2011), and the V363I and G304S mutations have been reported in cases with avPPA (Munoz et al., 2007; Villa et al., 2011).

As with *GRN* mutations, there is no clear correlation between the type of mutation and the clinical presentation; however, anecdotal observations suggest that exonic mutations that do not affect the splicing of exon 10 are usually associated with a dementia-predominant phenotype, and that intronic and exonic mutations that affect exon 10 splicing, leading to an overproduction of 4R tau, tend to be associated with a parkinsonism-predominant phenotype (Ghetti et al., 2015).

In some cases, episodic memory impairment has been observed in patients with *MAPT* mutations, in particular in the R406W mutation, mimicking Alzheimer’s disease (AD) (Tolboom et al., 2010), or in the recently described duplication of the *MAPT* gene, where the predominant memory impairment was preceded by clinical symptoms characteristic of bvFTD (Rovelet-Lecrux et al., 2010).

Neuroimaging studies have observed heterogeneous patterns of cortical atrophy, but with predominant involvement of the frontal and temporal lobes, with occasional involvement of the parietal lobes (Van Swieten et al., 1999; Janssen et al., 2002; Boeve et al., 2005; Ghetti et al., 2008; Whitwell et al., 2009a,b). The atrophy is particularly prominent in the anteromedial temporal lobes (Spina et al., 2008; Whitwell et al., 2009a,c; Rohrer et al., 2010b), with some studies showing that anteromedial atrophy is a feature of early stages of disease (Boeve et al., 2005). In the majority of individuals, patterns of cortical atrophy are relatively symmetrical (Van Swieten et al., 1999; Ghetti et al., 2008; Rohrer et al., 2011a); however, asymmetry has been observed in some cases (Boeve et al., 2005).

Atrophy of the basal ganglia and of the brainstem have been observed in studies on individual patients, possibly correlating with the parkinsonian features frequently associated with the disease (Arima et al., 2000; Soliveri et al., 2003; Slowinski et al., 2009).

In spite of the fact that temporal lobe atrophy is the most typical finding in patients with *MAPT* mutations, the pattern of cortical atrophy can differ depending on the specific mutation. Indeed *MAPT* mutations affecting the splicing of exon 10 are mainly associated with medial temporal atrophy, while mutations in the coding region are primarily associated with lateral temporal lobe atrophy (Whitwell et al., 2009a; Ghetti et al., 2015). These findings, if confirmed in other cohorts of subjects, could help identifying the underlying *MAPT* mutation.

As in structural imaging, functional imaging with F-18 fluorodeoxyglucose positron emission tomography (FDG-PET) often shows reduced frontal and/or temporal hypometabolism, which resembles the patterns observed in sporadic FTD (Frank et al., 2007).

As in *GRN* mutations, structural imaging changes can be identified years before expected onset of symptoms in asymptomatic adults at risk of genetic FTD. Rohrer et al. observed differences, between *MAPT* mutation carriers and non-carriers, in the hippocampus and amygdala 15 years before expected onset, followed by the temporal lobe 10 years before expected onset, and in the insula 5 years before expected onset (Rohrer et al., 2015b).

Functional connectivity in the default mode network (DMN) is altered in *MAPT* subjects before the occurrence of both atrophy and clinical symptoms, suggesting that changes in functional connectivity are early features of the disease. Asymptomatic *MAPT* subjects show reduced connectivity in the DMN predominantly between precuneus and lateral temporal lobe, but also medial prefrontal cortex, compared to age-matched controls (Whitwell et al., 2011a). Significantly decreased fractional anisotropy (FA) and increased radial diffusivity in bilateral uncinate fasciculi and reduced FA in the forceps minor have also been observed in asymptomatic *MAPT* carriers (Dopper et al., 2014).

A preliminary study with the F-18-T807 PET ligand for tau in a patient carrying a *MAPT* mutation has demonstrated a classic frontotemporal distribution characteristic of inherited tauopathies (Ghetti et al., 2015). These ligands, which characterize tau pathology *in vivo* (Fodero-Tavoletti et al., 2011; Xia et al., 2013), will undoubtedly improve our future understanding of the pathogenesis of disorders characterized by tau deposition (Villemagne et al., 2015).

## *GRN* Mutations

Since 2006, 69 distinct pathogenic mutations have been identified in the *GRN* gene, accounting for up to 20% of familial and 5% of sporadic FTD cases (Cruts et al., 2012). Progranulin, which is expressed in many cell types, including neurons and microglia, is a secreted growth factor known for its role in biological processes, including cellular and tissue development, inflammation, wound healing, and cancer, and for its neurotrophic properties. It is proteolytically processed into peptides called granulins, the function of which, in the nervous system, is still largely speculative (Bateman and Bennett, 2009).

Almost all pathogenic mutations cause disease by haploinsufficiency (Baker et al., 2006; Cruts et al., 2006), reducing the levels of progranulin in cerebrospinal fluid, plasma, and serum in symptomatic and asymptomatic *GRN* mutation carriers (Ghidoni et al., 2008; Finch et al., 2009; Sleegers et al., 2009). Indeed, the measurement of plasmatic progranulin levels is now largely used to screen patients for *GRN* sequencing (Schofield et al., 2010; Almeida et al., 2013).

A number of factors that influence progranulin expression have been identified in patients carrying *GRN* mutations. Genetic variants in T*MEM106B* (Van Deerlin et al., 2010), a gene encoding for the still uncharacterized transmembrane protein 106B, seem to delay the onset of disease in *GRN* mutation carriers, perhaps increasing the levels of progranulin (Cruchaga et al., 2011; Finch et al., 2011; van der Zee et al., 2011). Granulin expression is also regulated by microRNAs, including miR-29b and miR-107 (Jiao et al., 2010; Wang et al., 2010), and by sortilin (SORT1), a receptor for neurotrophic factors, which mediates progranulin endocytosis and regulates the plasmatic levels of progranulin (Carrasquillo et al., 2010; Hu et al., 2010).

The clinical presentation in patients with *GRN* mutations is highly variable, and phenotypic heterogeneity can be observed in the same family carrying an identical pathogenic mutation (Beck et al., 2008; Gabryelewicz et al., 2010; Yu et al., 2010; Chen-Plotkin et al., 2011), with an age-dependent and incomplete penetrance (Cruts and Van Broeckhoven, 2008). Age at symptom onset varies significantly, ranging from 35 to 89 years, with an average of 65 years, and a penetrance of 50% by the age of 60 and 90% by the age of 70 years (Gass et al., 2006; van Swieten and Heutink, 2008; Le Ber, 2013).

Within families, there is considerable variation in the age of onset, with a difference of up to 20 years between consecutive generations (Seelaar et al., 2008; van Swieten and Heutink, 2008). Neither age of disease onset nor the duration of the disease seems to have any correlation with the type or location of the *GRN* mutation.

The initially reported and most common clinical diagnosis in patients carrying *GRN* mutations is bvFTD, with significant apathy and social withdrawal (Baker et al., 2006; Cruts et al., 2006). In addition, subsequent studies identified, at onset, a clinical presentation characterized by aphasic disorders consistent with avPPA (Snowden et al., 2006; Mesulam et al., 2007; Le Ber et al., 2008), or CBS (Masellis et al., 2006). The aphasic disorder associated with *GRN* mutations seems nevertheless to be distinct from the avPPA with apraxia of speech observed occasionally in patients with FTLD-tau (Rohrer et al., 2010a,c), or from the logopenic variant of PPA (lvPPA), commonly associated with an atypical presentation of AD pathology (Gorno-Tempini et al., 2011; Rohrer et al., 2012).

Extrapyramidal features have been reported in about 40% of patients with *GRN* mutations and include CBS with limb apraxia, asymmetrical parkinsonism, and dystonia, although they are rarely a primary symptom and usually emerge after the onset of behavioral or language symptoms (Yu et al., 2010), and only occasionally respond to levodopa (Carecchio et al., 2009; Di Fabio et al., 2010).

In almost 25% of patients hallucinations and delusions can be predominant, frequently causing a misdiagnosis of dementia with Lewy bodies (DLB) (Le Ber et al., 2008; Kelley et al., 2010; Yu et al., 2010).

Furthermore, an AD-like phenotype can be observed in patients with episodic memory deficit, which occurs in 10–30% of cases, or with parietal deficits, such as dyscalculia, visuospatial symptoms, and limb apraxia. This phenotype can be occasionally associated with the c.154delA mutation in *GRN* (Kelley et al., 2010) or with the genetic variant rs5848, where the minor T allele, located in the 3′-untranslated region of *GRN*, was found to increase the binding of miR-659 to *GRN* (Brouwers et al., 2008; Rademakers et al., 2008; Lee et al., 2011; Sheng et al., 2014), consequently reducing serum progranulin levels (Hsiung et al., 2011).

A recent meta-analysis studied the association between the *GRN* polymorphism rs5848 and risk of neurodegenerative diseases, including FTLD, AD, Parkinson’s disease (PD), and ALS, concluding that rs5848 was associated with an increased risk of AD and PD in the homozygous and recessive models but not with FTLD (Chen et al., 2015).

Since *GRN* mutations are associated with FTD-TDP pathology, which is frequently observed also in ALS and FTD-ALS, it prompted the search for *GRN* mutations in patients with associated motor neuron disease. One study identified a missense mutation in a patient with FTD-ALS (p.S120Y) and in a single case of limb onset sporadic ALS (p.T182M) (Schymick et al., 2006), while a later study identified clinical features compatible with MND in 5.4% of patients carrying a *GRN* mutation; in this cohort, concomitant motor neuron disease was much more common in patients with FTD-TDP pathology not bearing a *GRN* mutation (26.3%) (Chen-Plotkin et al., 2011).

Recently, it has been shown that a complete *GRN* deficiency due to a homozygous *GRN* loss-of-function mutation causes the rare lysosomal storage disease neuronal ceroid lipofuscinosis (Smith et al., 2012). These findings suggest that lysosomal storage disorders and *GRN*-associated FTLD may share common features (Götzl et al., 2014).

Neuroimaging studies have highlighted that patients carrying a *GRN* mutation are more likely to show a pronounced asymmetrical atrophy (Huey et al., 2006; Bronner et al., 2007; Beck et al., 2008; Pickering-Brown et al., 2008) affecting either the left or right hemispheres, involving primarily the inferior frontal (Bronner et al., 2007; Beck et al., 2008; Ghetti et al., 2008), temporal and inferior parietal lobes (Mesulam et al., 2007; Pickering-Brown et al., 2008), as well as long intrahemispheric association white matter (WM) tracts (Borroni et al., 2008; Rohrer and Warren, 2011). A prominent feature, which is unusual in FTLD, is the predominance of parietal involvement (Whitwell et al., 2007; Beck et al., 2008; Le Ber et al., 2008), recently confirmed also in presymptomatic mutation carriers (Rohrer et al., 2008, 2015b; Cruchaga et al., 2009). These findings have been also confirmed by voxel-based morphometry (VBM) studies, which detected a widespread and severe pattern of gray matter loss predominantly affecting the frontal, temporal, and parietal lobes (Whitwell et al., 2009b, 2012). Along with cortical involvement, atrophy of deep subcortical structures has been identified in patients carrying *GRN* mutations, in particular in the caudate nucleus and in the thalamus bilaterally, possibly correlating with the parkinsonian features that are frequently associated since the early stages of the disease (Premi et al., 2014b).

Furthermore, cerebellar atrophy can be observed in some cases (Nuytemans et al., 2007; Benussi et al., 2008).

Subcortical WM involvement, ranging from T2-weighted MRI hyperintensities (Nuytemans et al., 2007; Le Ber et al., 2008; Kelley et al., 2009) to widespread WM lesions may be linked to progranulin pathological processes in a subset of *GRN* mutation carriers (Caroppo et al., 2014).

Annualized rates of whole brain atrophy, and consequently of ventricular expansion, are greatest in *GRN*, followed by sporadic FTD, *C9ORF72*, and lastly *MAPT* (Whitwell et al., 2015), in which similar rates of hippocampal atrophy have been observed (Whitwell et al., 2011b).

Using single photon emission computed tomography (SPECT), hypoperfusion of the hippocampus and that of the cingulate gyrus have been observed, denoting the association of these regions with the pathogenesis of *GRN* mutations (Le Ber et al., 2008).

Recent studies have highlighted that structural imaging and cognitive changes can be identified years before expected onset of symptoms in asymptomatic adults at risk of genetic FTD. In the study by Rohrer et al., in the *GRN* group, differences were observed between carriers and non-carriers in the insula at 15 years before expected onset, then in the temporal and parietal lobes at 10 years before expected onset, with the earliest subcortical area affected being the striatum at 5 years before expected onset. *GRN* mutation carriers also showed significantly greater asymmetry than non-carriers at 5 years before expected onset (Rohrer et al., 2015b).

In functional connectivity studies, Premi et al. observed in asymptomatic carriers of the *GRN* Thr272fs mutation, decreased brain connectivity within the left frontoparietal network and increased connectivity in the executive network compared with healthy controls (Premi et al., 2013b; 2014a). Furthermore, the *TMEM106B* at-risk polymorphism (T/T) was associated with decreased connectivity within the ventral salience network (i.e., middle frontal gyrus) and the left frontoparietal network (i.e., left precuneus) (Premi et al., 2013a). Further studies have confirmed the patterns of brain atrophy and WM tract abnormalities in frontal-parietal circuits and within the uncinate fasciculus, supporting the hypothesis that WM damage is an early feature in the FTD disease process and can be detected at least a decade before the estimated symptom onset in asymptomatic mutation carriers (Dopper et al., 2014; Pievani et al., 2014).

## *C9orf72* Mutations

The strong association between FTD and ALS has become increasingly apparent due to a rapidly expanding body of clinical, epidemiological, genetic, and molecular evidence (Gros-Louis et al., 2006) and, in 2006, a locus was mapped on chromosome 9p21 in FTD-ALS families (Vance et al., 2006). Until 2011, despite numerous efforts, the disease mutation remained elusive (Momeni et al., 2006; Morita et al., 2006; Valdmanis et al., 2007; Luty et al., 2008; Le Ber et al., 2009; Boxer et al., 2011; Pearson et al., 2011). Eventually, DeJesus-Hernandez and colleagues observed the non-Mendelian inheritance of a GGGGCC hexanucleotide repeat, located in a non-coding region of the *C9orf72* gene, in the large VSM-20 family (Vancouver, San Francisco and Mayo family 20) affected by FTD-ALS, with the intuition that the repeat expansion, which was identified in the parents but not in the affected children by using fluorescent PCR, was too large to be amplified by the PCR method and could only be identified by repeated-primed PCR assay and Southern blot analysis (DeJesus-Hernandez et al., 2011). With the use of next-generation sequencing, another group independently recognized the repeat expansion in a Welsh family affected by FTD-ALS (Renton et al., 2011).

Until recently, C9orf72 was a completely uncharacterized protein with unknown function. The pathogenesis underlying the *C9orf72* mutation still remains unclear, but likely includes haploinsufficiency through loss of gene expression (Gijselinck et al., 2012; Cruts et al., 2013; van der Zee et al., 2013), gain-of-function mechanisms with secondary RNA toxicity caused by sequestration of RNA-binding proteins (DeJesus-Hernandez et al., 2011; Lagier-Tourenne et al., 2013), or both (Rohrer et al., 2015a).

Two different protein isoforms of the *C9orf72* gene are predicted to be generated from a total of three different transcripts variants, depending on the localization of the GGGGCC repeat, either in the promoter or in the intron 1 of the gene (DeJesus-Hernandez et al., 2011). All three different transcripts have been found to be reduced in blood (Mori et al., 2013a; Davidson et al., 2014), cortex, cerebellum, and spinal cord (Belzil et al., 2013; Donnelly et al., 2013). This reduced expression possibly reflects, to a limited extent, the hypermethylation of a CpG island upstream of the repeat (Xi et al., 2013; Belzil et al., 2014; Liu et al., 2014), and the methylation of lysine residues on H3 and H4 histones, which bind the GGGGCC repeats and suppress transcription (Xi et al., 2013; Davidson et al., 2014; Russ et al., 2014), acting as a possible innate protective mechanism (Liu et al., 2014; McMillan et al., 2015; Xi et al., 2015).

As previously mentioned, the neuropathological hallmarks, associated with *C9orf72* mutations, are the TDP-43 immunoreactive inclusions, i.e., subtypes A and B (Mahoney et al., 2012; Lashley et al., 2014), which have been observed in many cerebral structures, including the extramotor cerebral cortex, hippocampus, basal ganglia, substantia nigra, and lower motor neurons of the brainstem and spinal cord (Rademakers et al., 2012). In addition to the TDP-43 pathology, a distinctive and singular feature is the presence of abundant TDP-43 negative, p62-positive inclusions, with a unique star-like morphology, in the cerebellum, hippocampus, and frontal cortex (Al-Sarraj et al., 2011; Simon-Sanchez et al., 2012; Snowden et al., 2012; Troakes et al., 2012; Mori et al., 2013b).

Recently, Mori and colleagues tried to characterize these inclusions, speculating that they may consist of dipeptide repeat (DPR) proteins which are the result of the bidirectional translation of the non-coding GGGGCC expansion repeat through a mechanism of non-ATG-initiated translation, termed RAN translation (Mori et al., 2013c). Indeed, the researchers demonstrated that the GGGGCC expansion is bidirectionally translated into five distinct DPR proteins (Mori et al., 2013c), which have been observed in high load in neocortical regions, hippocampus, cerebellum and, to some extent, in motor neurons (Mackenzie et al., 2013). Moreover, the recent observation, in a Belgian *C9orf72* carrier, of neuropathological aggregates of a DPR core surrounded by TDP-43 inclusions, suggested that DPR aggregation due to RAN translation could be the initial pathological event that possibly precedes and potentially triggers TDP-43 accumulation (Mackenzie et al., 2013; Mori et al., 2013c; van der Zee and Van Broeckhoven, 2014).

These findings have led to the suggestion that *C9orf72* expansion cases should be reclassified, from a neuropathological prospective, as FTLD-DPR, since identified cases can fit into one of two FTLD-TDP subtypes (A or B), or might even lack TDP-43 pathology (Snowden et al., 2012; Mori et al., 2013c).

Despite these new findings implicating both loss-of-function and gain-of-function mechanism with new insights into the roles of sense (GGGGCC) and antisense (GGCCCC) repeat RNA and DPR proteins, the relative contribution to these mechanisms and the molecular pathways that lead to neurodegeneration are yet to be elucidated (Rohrer et al., 2015a).

The number of repeat units of the *C9orf72* expansion in affected patients can range from at least several hundred to thousands of copies, whereas unaffected healthy controls harbor 2–24 repeat units (DeJesus-Hernandez et al., 2011; Beck et al., 2013; van Blitterswijk et al., 2013). So far, there is no clear evidence for intergenerational anticipation, although some studies have identified earlier disease onset in subsequent generations (Stewart et al., 2012; Benussi et al., 2014).

Whilst it is now rather clear that expansions of several hundred copies are definitely pathogenic, it is not known which is the smallest hexanucleotide expansion that can confer a risk of developing the disease. Indeed, *in vitro* studies have demonstrated an inverse correlation between repeat size and gene expression starting at just 9 repeats, with a reduction of up to 50% in expression for 24 expansion repeats (van der Zee et al., 2013).

Furthermore, as in other non-coding repeat expansion disorders, evidence for somatic instability of the *C9orf72* repeat has been observed (DeJesus-Hernandez et al., 2011), resulting in a range of mutation sizes in particular tissues with variation between tissues in the same individual. This possibly suggest that intermediate repeats detected in blood-sample-derived DNA might be associated with massive expansions in the brain (Rohrer et al., 2015a), making it even harder to determine correlations between genetics, phenotype, and neuropathology (Lavedan et al., 1993; Matsuura et al., 2004). Indeed, a recent study tried to estimate, with Southern blotting techniques and densitometry, the repeat size of the most abundant GGGGCC expansion species in patients with ALS and FTD-ALS. Comparing different tissues, substantial variation in repeat sizes between samples from the cerebellum, frontal cortex, and blood have been identified. In particular, longer repeat sizes in the cerebellum seem to be associated with a survival disadvantage, indicating that expansion size does affect disease severity (Majounie et al., 2012).

As with *GRN* mutations, *TMEM106B* has been identified as a genetic modifier also in *C9orf72* cases, demonstrating a significantly later age at death and age at onset for the rs1990622 major allele (T) carriers (Gallagher et al., 2014).

Since the discovery of the *C9orf72* mutation, testing of patients’ cohorts worldwide has found an average frequency of the pathogenic expansion in 37% of familial ALS, 6% of sporadic ALS, 21% of familial FTD, and 6% of sporadic FTD. Epidemiological studies have all established that the *C9orf72* mutation is the major genetic cause of familial ALS (more frequent than superoxidose dismutase 1 mutations), and comparable in frequency to *GRN* mutations in familial FTD cases (DeJesus-Hernandez et al., 2011; Renton et al., 2011; Chiò et al., 2012; Hsiung et al., 2012; Majounie et al., 2012; Cruts et al., 2013; van der Zee et al., 2013).

Age at disease onset is widely variable, ranging from 27 to 83 years (mean 50 years), with about 50% patients affected by age 60 and with a penetrance reaching 90% at age 70. Disease duration is also variable, ranging from 1 to 22 years, with the development of ALS as a strong negative prognostic factor (Boeve et al., 2012; Chiò et al., 2012; Cooper-Knock et al., 2012; Gijselinck et al., 2012; Hsiung et al., 2012; Stewart et al., 2012).

Clinical phenotype is highly heterogeneous and variable even within families affected by the same mutation. The most common clinical presentations are FTD (Boeve et al., 2012; Dobson-Stone et al., 2012; Englund et al., 2012; Hsiung et al., 2012; Mahoney et al., 2012; Sha et al., 2012; Snowden et al., 2012; Devenney et al., 2014), ALS (Boeve et al., 2012; Byrne et al., 2012; Chiò et al., 2012; Cooper-Knock et al., 2012; Stewart et al., 2012; Coon et al., 2013; Montuschi et al., 2015), or the combination of both. The most frequent FTD subtype is bvFTD, with avPPA observed less frequently, although other phenotypes have been described, and even cases with classical bvFTD or ALS have extensive phenotypic heterogeneity. bvFTD associated with the *C9orf72* mutation may present with classical features, as apathy, disinhibition, socially inappropriate behavior, abnormal eating behavior, and loss of empathy (Rascovsky et al., 2011). Nevertheless, behavioral changes with features of psychosis, including hallucinations and delusions, are not rarely observed in these patients (Arighi et al., 2012; Boeve et al., 2012; Dobson-Stone et al., 2012; Englund et al., 2012; Mahoney et al., 2012; Snowden et al., 2012; Galimberti et al., 2013; Kertesz et al., 2013; Devenney et al., 2014), and can even predominate at disease onset and lead to an initial diagnosis of schizophrenia (Galimberti et al., 2014a), bipolar disorder (Galimberti et al., 2014b), obsessive–compulsive disorder (Calvo et al., 2012), depressive pseudodementia (Bieniek et al., 2014), or DLB (Robinson et al., 2014).

Mutations in *C9orf72* have been rarely observed in patients with PPA (Renton et al., 2011; Dobson-Stone et al., 2012; Englund et al., 2012; Mahoney et al., 2012; Sha et al., 2012; Simon-Sanchez et al., 2012; Snowden et al., 2012; Van Langenhove et al., 2013; Saint-Aubert et al., 2014).

As in *GRN* and *MAPT* mutations, deficits in episodic memory at onset, mimicking AD have been reported also in patients with *C9orf72* mutations (Wojtas et al., 2012; Cacace et al., 2013; Harms et al., 2013; Murray et al., 2013; Pletnikova et al., 2014). Moreover, extrapyramidal movement disorder (usually an akinetic-rigid syndrome) (Murray et al., 2011; Boeve et al., 2012; Cooper-Knock et al., 2012; Floris et al., 2012; Hsiung et al., 2012; Simon-Sanchez et al., 2012) and cerebellar signs have also been reported (Hsiung et al., 2012). Most patients, during the course of the disease, eventually develop some abnormalities of behavior, language, and motor function (Boeve, 2011; Byrne et al., 2012; Cooper-Knock et al., 2012; Hsiung et al., 2012; Stewart et al., 2012).

Structural neuroimaging in patients with *C9orf72* expansions does not reveal a characteristic pattern of atrophy, unlike *GRN* or *MAPT* mutations. A prevalently symmetrical, bilateral, cortical, and subcortical atrophy, predominantly affecting the frontotemporal regions, has been observed (Boeve et al., 2012; Dobson-Stone et al., 2012; Englund et al., 2012; Mahoney et al., 2012; Sha et al., 2012; Snowden et al., 2012; Whitwell et al., 2012; Devenney et al., 2014).

Neuroimaging studies using VBM analysis have observed extensive atrophy of the frontal, insular, temporal, and parietal cortical and subcortical regions, including prominent atrophy of the thalamus and cerebellum (Mahoney et al., 2012; Whitwell et al., 2012; Irwin et al., 2013).

Concordant with gray matter atrophy, WM atrophy of long intrahemispheric, commissural, and corticospinal tracts has been observed with diffusion tractography (Mahoney et al., 2012).

As with structural neuroimaging, functional neuroimaging using FDG-PET and SPECT has shown frontal, temporal, and parietal cortical and subcortical alterations (Boeve et al., 2012; Adeli et al., 2014; Suárez-Calvet et al., 2014). Seed-based intrinsic connectivity analyses in *C9orf72* carriers versus non-carriers showed connectivity reductions in the salience and sensorimotor networks, with severity of behavioral symptom correlating with diminished salience network connectivity and increased DMN connectivity (Lee et al., 2014).

As in *GRN* and *MAPT* mutations, presymptomatic imaging signatures can be identified in asymptomatic adults at risk of genetic FTD. Rohrer et al. observed differences, between *C9orf72* mutation carriers and non-carriers, in subcortical areas, including the thalamus, the insula, and posterior cortical areas 25 years before expected onset, followed by the frontal and temporal lobes 20 years before expected onset, and in the cerebellum 10 years before expected onset (Rohrer et al., 2015b).

## *VCP* Mutations

Missense mutations in the *VCP* gene, located on chromosome 9p13.3, were originally reported in 2004 in North American families with a specific autosomal dominant multisystem syndrome called inclusion body myopathy with Paget disease of bone and/or FTD (IBMPFD) (Watts et al., 2004), with neuropathology consistent with type D TDP-43 pathology (van der Zee et al., 2009).

Valosin-containing protein, also known as p97, is a member of the ATPases associated with various cellular activities (AAA) family of proteins and is involved in multiple cellular processes, including protein degradation via both proteasome and autophagy pathways, in membrane fusion, transcriptional activation, apoptosis, and molecular chaperoning (Nalbandian et al., 2011; Yamanaka et al., 2012). The role of mutant VCP in the pathogenesis of IBMPFD or FTD-ALS remains elusive.

So far, 19 pathogenic mutations in *VCP* have been described (Cruts et al., 2012). The frequency of *VCP* mutations in FTD is about 1.6%, making it a rare but significant cause of FTD (van der Zee et al., 2009). Nearly 80% of *VCP* mutation carriers have a positive family history with a wide variability in phenotypic presentation, even within the same family.

The disease usually begins at the age of 40 with musculoskeletal symptoms not dissimilar to limb-girdle muscular dystrophy, affecting more than 80% patients. Moreover, nearly 60% patients develop adult-onset Paget disease of bone (Watts et al., 2004). FTD usually develops later on in the disease course (age 55–60 years), affecting about 30% of patients, with a classical bvFTD phenotype (Kovach et al., 2001; Guyant-Marechal et al., 2006; Kimonis et al., 2008); occasional psychiatric disorders have been reported (Jacquin et al., 2013). However, although rare, *VCP* mutations have been found in patients with a dementia-only phenotype (van der Zee et al., 2009). Early language deficits and semantic impairment have been rarely described (Kim et al., 2011).

*VCP* mutations have been identified also in patients with an MND/ALS phenotype, occasionally associated with FTD (Johnson et al., 2010; Miller et al., 2012; Hirano et al., 2015); these findings are supported by the pyramidal tract dysfunction which has been observed in some cases (Kumar et al., 2010).

The term multisystem proteinopathy has been recently proposed to replace the term IBMPFD to describe this phenotype (Benatar et al., 2013).

MRI findings range widely, from cases showing no signs of frontal lobe atrophy to patients with prominent, asymmetric, focal atrophy in the anterior and temporal lobes (Gidaro et al., 2008; Djamshidian et al., 2009; Stojkovic et al., 2009; Kim et al., 2011; Surampalli et al., 2015). FDG-PET imaging has revealed severe glucose hypometabolism in the bilateral frontotemporoparietal areas (Guyant-Marechal et al., 2006; Stojkovic et al., 2009; Kim et al., 2011).

## *TARDBP* Mutations

Transactive response DNA-binding protein 43 (TDP-43) is a ubiquitously expressed and highly conserved ribonucleoprotein, encoded by the *TARDBP* gene on chromosome 1, with a putative role in the regulation of transcriptional activity, of messenger RNA splicing, exon skipping, and microRNA biogenesis of several genes. It has been proposed that, in neurodegenerative disorders, TDP-43 can be redistributed from the nucleus to the cytoplasm and sequestered into inclusions that are mainly composed of abnormally phosphorylated and C-terminally truncated TDP-43 fragments (Buratti and Baralle, 2008; Polymenidou et al., 2011; Tollervey et al., 2011).

The identification of mutations within the *TARDBP* gene as causative for both familial and sporadic ALS provided additional proofs for the pathogenic role of TDP-43 in neurodegeneration (Kabashi et al., 2008; Rutherford et al., 2008; Sreedharan et al., 2008; Van Deerlin et al., 2008).

Mutations in the *TARDBP* gene were initially described in 2008 in patients with ALS, both in familial and sporadic cases (Kabashi et al., 2008; Sreedharan et al., 2008; Van Deerlin et al., 2008). So far, mutations in the *TARDBP* gene account for 2–3% of ALS patients (Gitcho et al., 2008; Kabashi et al., 2008; Kühnlein et al., 2008; Rutherford et al., 2008; Sreedharan et al., 2008; Van Deerlin et al., 2008; Yokoseki et al., 2008; Daoud et al., 2009).

Although previous studies failed to identify a significant role for *TARDBP* mutations in FTD (Rollinson et al., 2007; Gallone et al., 2009; Gijselinck et al., 2009; Schumacher et al., 2009), recently it has been reported that *TARDBP* mutations might be causative of FTD with MDN. These uncommon cases present with a heterogeneous phenotype, almost always associated with a significant motor neuron involvement, including FTD-MND (Benajiba et al., 2009; Chiò et al., 2010), FTD-MND with extrapyramidal symptoms (Borghero et al., 2011; Chiò et al., 2011; Quadri et al., 2011; Mosca et al., 2012), and MND with supranuclear palsy (Kovacs et al., 2009; Borghero et al., 2011). Pure FTD cases without motor neuron involvement carrying a TARDBP mutation are even more exceptional. Indeed, less than 16 cases have been reported so far (Borroni et al., 2009, 2010; Gitcho et al., 2009; Kovacs et al., 2009; Gelpi et al., 2014; Synofzik et al., 2014; Moreno et al., 2015), with only four cases having neuropathological confirmation (Gitcho et al., 2009; Kovacs et al., 2009; Gelpi et al., 2014; Moreno et al., 2015). The most common phenotype that was described was bvFTD followed by PPA, in particular with svPPA, with a heterogeneous age of onset, ranging from 29 to 77 years.

Neuroimaging studies, achieved only in case reports and small series, show predominant temporal lobe involvement (Borroni et al., 2009, 2010; Gitcho et al., 2009; Kovacs et al., 2009; Gelpi et al., 2014; Synofzik et al., 2014; Moreno et al., 2015).

TDP-43 inclusions, as previously outlined, have been observed also in non-*TARDBP*-associated genetic forms of FTLD, including *GRN*, *VCP*, and *C9orf72* mutations, suggesting that dysregulation of TDP-43 might be a pivotal downstream pathogenic mechanism involved in neurodegeneration in each of these disorders (Rademakers et al., 2012).

## *CHMP2B* Mutations

The *CHMP2B* gene, located on chromosome 3p11.2, encodes the charged multivesicular body protein 2B, which has been identified in 2005 in a large Danish family with an autosomal form of FTLD (Skibinski et al., 2005), first described in 1987 (Gydesen et al., 1987). The average age of onset in this family was 55 years, ranging from 46 to 65 years. Since 2005, researchers identified *CHMP2B* missense variants in FTD and FTD-ALS cases, representing less than 1% of familial forms of FTLD, although the pathogenicity of these variants remain to be elucidated (Parkinson et al., 2006; Cox et al., 2010).

The phenotypic expression of the originally described family comprises bvFTD that may be associated with late parkinsonism, dystonia, myoclonus, and pyramidal symptoms (Skibinski et al., 2005).

Neuropathological examination revealed ubiquitin/p62-positive, TDP-43/FUS negative neuronal inclusions, termed FTLD-U (Holm et al., 2007, 2009).

## *FUS* Mutations

The genetic heterogeneity of FTD is further emphasized by the occurrence of rare mutations in the *FUS* gene, located on chromosome 16, which was first identified as a fusion oncogene in human liposarcomas (Crozat et al., 1993; Rabbitts et al., 1993).

FUS, as TAF-15 and EWS, belongs to the FET family of DNA/RNA-binding proteins, which are highly conserved, ubiquitously expressed, multifunctional binding proteins (Tan and Manley, 2009) that can bind to a large number of overlapping RNA targets (Hoell et al., 2011). Although the precise physiological function of FUS has not been fully elucidated, growing evidence suggests that the protein is involved in various cellular processes, including cell proliferation, DNA repair, transcription regulation, and multiple levels of RNA and microRNA processing (Lagier-Tourenne et al., 2010; Deng et al., 2014).

In 2009, *FUS* gene mutations were identified as the primary disease-associated gene for familial (Kwiatkowski et al., 2009; Vance et al., 2009; Doi et al., 2010) and sporadic ALS (Belzil et al., 2009). This mutation accounted for nearly 3% of familial ALS cases, characterized by FUS-positive, TDP-43 negative inclusions (Kwiatkowski et al., 2009; Vance et al., 2009), consequently termed ALS-FUS. Eventually, FUS was found to be the most characteristic marker of the remaining tau-negative and/or TDP-43 negative FTLD cases, encompassing three closely related but distinct clinicopathological entities: atypical FTLD-U (aFTLD-U), neuronal intermediate filament inclusion disease (NIFID), and basophilic inclusion body disease (BIBD) (Munoz et al., 2009; Neumann et al., 2009a,b; Urwin et al., 2010).

*FUS* mutations in patients with clinical FTLD are rare, and only four *FUS* mutations have so far been identified in patients with FTD-MND/ALS or in their families (Ticozzi et al., 2009; Blair et al., 2010; Broustal et al., 2010; Yan et al., 2010). Moreover, two cases with a pure FTD phenotype without motor neuron involvement have also been reported (Van Langenhove et al., 2010; Huey et al., 2012). Nevertheless, no autopsy data are yet available to confirm FUS pathology in any patient with FTLD and *FUS* mutations.

In contrast to ALS with FUS pathology, which is mostly caused by *FUS* mutations, FTLD-FUS tends to be sporadic, with none yet associated with a mutation in the *FUS* gene (Rademakers et al., 2012).

Different pathogenic mechanisms in ALS-*FUS* and FTLD-FUS also arise from a recent study in which, in ALS-*FUS*, no co-accumulation of other FET-proteins into FUS-positive inclusions was found, in remarkable contrast to FTLD-FUS, in which TAF-15 and EWS were found to co-accumulate in FUS-positive cytoplasmic inclusions (Neumann et al., 2011).

Structural neuroimaging studies show predominant volume loss in the anterior temporal lobe, amygdala, anterior hippocampus, anterior insula, and anterior cingulate cortex (Lee et al., 2012). Bilateral anterior striatal atrophy is particularly prominent in the head of the caudate nucleus, which appears to be significantly greater in FTLD-FUS compared with FTLD-TDP and FTLD-tau, suggesting that severe caudate atrophy may be a useful clinical feature to predict FTLD-FUS pathology (Josephs et al., 2010; Lee et al., 2012).

## Other Genetic Causes: *DCTN1* and *TREM2*

There are few reports of *DCTN1* mutations in patients with FDT and MND/ALS (Münch et al., 2005; Newsway et al., 2010), variably associated with parkinsonism, hypoventilation, dysautonomia, weight loss, and behavioral disturbances configuring the recently described Perry syndrome, which is associated with TDP-43 pathology (Farrer et al., 2009; Ohshima et al., 2010). Notwithstanding, no mutations were found in large FTD or MND/ALS cohorts (Seelaar et al., 2008; Vilariño-Güell et al., 2009).

Homozygous loss-of-function mutations in *TREM2* on chromosome 6p21, encoding the triggering receptor expressed on myeloid cells 2 protein, have been initially associated with an autosomal recessive form of early onset dementia presenting with bone cysts and consequent fractures called polycystic lipomembranous osteodysplasia with sclerosing leukoencephalopathy, also known as Nasu-Hakola disease (Paloneva et al., 2000, 2002).

In 2013, two studies identified a rare heterozygous variant (R47H) on the second exon of *TREM2*, showing an approximately three- to fivefold increased risk in late onset AD (Guerreiro et al., 2013c; Jonsson et al., 2013). Furthermore, homozygous and compound heterozygous *TREM2* exon 2 mutations were identified in rare families presenting with autosomal recessive bvFTD without any clinical or radiological evidence of bone involvement (Giraldo et al., 2013; Guerreiro, 2013; Guerreiro et al., 2013a; Lattante et al., 2013; Rayaprolu et al., 2013; Le Ber et al., 2014). Patients typically have very young age at onset (20–50 years) and, in addition to behavioral changes, they present with early parietal and hippocampal deficits, extrapyramidal features, and seizures. Neuroimaging frequently shows frontotemporal atrophy and extensive WM abnormalities (Guerreiro, 2013; Le Ber et al., 2014).

Recently, Borroni and colleagues evaluated the frequency of heterozygous *TREM2* mutations as risk factor in FTD patients, confirming that the Q33X, R47H, and T66M mutations were significantly associated with the disease and absent in healthy subjects (Borroni et al., 2013), in line with further reports (Cuyvers et al., 2013).

## Conclusion

The striking clinical, genetic, and pathological heterogeneity are undisputable features of FTLD. The clinical phenotypes range from social to language impairment, variably associated with MND/ALS or extrapyramidal symptoms (see Table 1). In the past 9 years, considerable progress has been made toward unraveling the genetic causes of FTLD, with apparently all common FTD-related genes discovered, comprising *C9orf72*, *MAPT*, *GRN*, *TARDBP*, *VCP*, *CHMP2B*, and *FUS*, currently identifying 50–60% of the familial forms of FTD, although rare mutations in other genes could explain a small number of the remaining families (Rademakers et al., 2012; Le Ber, 2013).

**Table 1:** **Main clinical presentation in monogenic FTLD**.

Gene	Main clinical presentation				
	bvFTD	avPPA	svPPA	PSP/CBS	MND/ALS
*GRN*	+++	+++	−	++^a^	−
*MAPT*	++	+	−	++	−
*C9orf72*	+++	++	+	+	+++
*VCP*	+	−	−	−	+
*TARDBP*	+	−	+	−	++
*CHMP2B*	+	−	−	+	+
*FUS*	+	−	−	−	++
*DCTN1*	−	−	−	+	+
*TREM2*	+	−	−	+	−

*Semi-quantitive grading: +++, very frequent; ++, frequent; +, infrequent; −, sporadic cases or no cases reported*.

*GRN, progranulin; *MAPT*, microtubule-associated protein tau; *C9orf72*, chromosome 9 open reading frame 72; *VCP*, valosin-containing protein; *TARDBP*, transactive response DNA-binding protein 43, *CHMP2B*, charged multivesicular body protein 2B; *FUS*, fused in sarcoma; *DCTN1*, dynactin; *TREM2*, triggering receptor expressed on myeloid cells; bvFTD, behavioral variant of frontotemporal dementia; avPPA, agrammatic variant of primary progressive aphasia; svPPA, semantic variant of primary progressive aphasia; PSP/CBS, progressive supranuclear palsy/corticobasal syndrome features; MND/ALS, motor neuron disease/amyotrophic lateral sclerosis*.

*^a^CBS presentation only*.

Neuropathology is variably associated with two predominant hallmarks, namely FTLD-tau (found in *MAPT* mutations) and FTLD-TDP (found in *GRN*, *C9orf72*, *TARDPB*, and *VCP* mutations), although it is occasionally associated with FTLD-FUS (found in *FUS* mutations only in MND/ALS), FTLD-U (found in *CHMP2B* mutations) and the newly identified FTLD-DPR in *C9orf72* mutations.

Why different genes linked to different molecular pathways are responsible for a neurodegenerative process involving mainly the frontotemporal lobes is still a matter for debate, and the shared mechanisms that lead to predominant frontal and temporal degeneration remain to be identified.

As previously outlined, frequently there is poor correspondence between the type of mutation or neuropathological feature and the clinical phenotype; indeed, neuropathology is predicable only in known genetic defects and few clinical or neuroanatomical features have a specific molecular association (Rohrer et al., 2011a). Both clinically and anatomically, different genetic mutations show substantial overlap between diseases. However, certain distinctive clinical and neuroimaging features emerge and might help in driving neurology practice in molecular diagnostics. Patients with *C9orf72* repeat expansion usually have an early disease onset and a family history of FTD (mainly bvFTD with disinhibition or svPPA), which is variably associated with MND/ALS. *MAPT* mutation carriers often present with a very early age of onset bvFTD, associated with parkinsonism, which is a common feature later in the disease course in all types of genetic FTLD. *GRN* mutations carriers frequently present with bvFTD, avPPA, and concomitant CBS at onset. *TARDBP* sequencing should be performed in familial or sporadic *C9orf72* negative FTD-MND/ALS cases. The association of inclusion body myopathy and Paget’s disease are indicative of *VCP* mutations (Rohrer and Warren, 2011; Borroni and Padovani, 2013; Le Ber, 2013; Van Langenhove et al., 2013).

In addition to these distinct clinical features, from a neuroimaging perspective, strikingly asymmetric interhemispheric atrophy with frontoparietal involvement is indicative of *GRN* mutations, while relative symmetric atrophy with particular involvement of the anterior temporal lobes is indicative of *MAPT* mutations (Rohrer et al., 2010b; Whitwell et al., 2012). On the other hand, *C9orf72* mutation carriers tend to have a more generalized and symmetrical atrophy (Rohrer et al., 2010b; Whitwell et al., 2012; Van Langenhove et al., 2013) (see Table 2).

**Table 2:** **Preclinical and clinical neuroanatomical correlates in monogenic FTLD**.

Gene	Severity of atrophy in preclinical/clinical phase					
	FL	TL	PL	IN	HC	ST
*GRN*	+/+++	+/++	+/++	++/+	−/−	−/−
*MAPT*	−/++	++/+++	−/−	+/+++	++/+++	−/+++
*C9orf72*	+++/+++	++/+++	++/++	+++/+++	+++/+++	−/−
*VCP*	−/++	−/++	−/+	−/−	−/++	−/−
*TARDBP*	−/+	−/++	−/+	−/+	−/−	−/−
*CHMP2B*	+/+	+/++	+/+	+/+	−/−	−/−
*FUS*	−/++	−/++	−/++	−/−	−/−	−/+++
*DCTN1*	−/++	−/++	−/+	−/+	−/++	−/−
*TREM2*	−/++	−/+	−/++	−/−	−/+	−/+

*Semi-quantitive grading: +++, severe; ++, moderate; +, mild; −, absent or not reported*.

*FL, frontal lobe; TL, temporal lobe; PL, parietal lobe; IN, insula; HC, hippocampus; ST, striatum; *GRN*, progranulin; *MAPT*, microtubule-associated protein tau; *C9orf72*, chromosome 9 open reading frame 72; *VCP*, valosin-containing protein; *TARDBP*, transactive response DNA-binding protein 43; *CHMP2B*, charged multivesicular body protein 2B; *FUS*, fused in sarcoma; *DCTN1*, dynactin; *TREM2*, triggering receptor expressed on myeloid cells*.

Numerous attempts have been made to identify an *in vivo* biomarker that can accurately predict the underlying neuropathology and consequently point out which gene should be analyzed, but a clear-cut association between clinical or neuroradiological features and characteristic histological inclusions has not been yet established (Kim et al., 2007; Josephs et al., 2008; McMillan et al., 2013). Plasma progranulin levels have been shown to be reliable predictors of *GRN* mutations, and should be performed before *GRN* sequencing (Finch et al., 2009; Ghidoni et al., 2012). Furthermore, the recently identified cerebrospinal phospho-tau_181_ to total-tau ratio appears to predict FTLD-TDP pathology with high accuracy, thus leading to the search of genes associated with TDP-43 neuropathology (Hu et al., 2013; Grossman et al., 2014; Borroni et al., 2015a).

Considering the vast clinical, genetic, and pathological heterogeneity associated with this disease, the development of accurate *in vivo* biomarkers is mandatory for guiding genetic screening, for defining distinctive therapeutic approaches and helping in patients’ selection in future clinical trials.

## Conflict of Interest Statement

The authors declare that the research was conducted in the absence of any commercial or financial relationships that could be construed as a potential conflict of interest.
